# Investigating International Time Trends in the Incidence and Prevalence of Atopic Eczema 1990–2010: A Systematic Review of Epidemiological Studies

**DOI:** 10.1371/journal.pone.0039803

**Published:** 2012-07-11

**Authors:** Ivette A. G. Deckers, Susannah McLean, Sanne Linssen, Monique Mommers, C. P. van Schayck, Aziz Sheikh

**Affiliations:** 1 Allergy and Respiratory Research Group, Centre for Population Health Sciences, The University of Edinburgh, Edinburgh, United Kingdom; 2 CAPHRI, Department of Epidemiology, Maastricht University Medical Centre+, Maastricht, The Netherlands; 3 CAPHRI, Department of General Practice, Maastricht University Medical Centre+, Maastricht, The Netherlands; The Australian National University, Australia

## Abstract

The prevalence of atopic eczema has been found to have increased greatly in some parts of the world. Building on a systematic review of global disease trends in asthma, our objective was to study trends in incidence and prevalence of atopic eczema. Disease trends are important for health service planning and for generating hypotheses regarding the aetiology of chronic disorders. We conducted a systematic search for high quality reports of cohort, repeated cross-sectional and routine healthcare database-based studies in seven electronic databases. Studies were required to report on at least two measures of the incidence and/or prevalence of atopic eczema between 1990 and 2010 and needed to use comparable methods at all assessment points. We retrieved 2,464 citations, from which we included 69 reports. Assessing global trends was complicated by the use of a range of outcome measures across studies and possible changes in diagnostic criteria over time. Notwithstanding these difficulties, there was evidence suggesting that the prevalence of atopic eczema was increasing in Africa, eastern Asia, western Europe and parts of northern Europe (i.e. the UK). No clear trends were identified in other regions. There was inadequate study coverage worldwide, particularly for repeated measures of atopic eczema incidence. Further epidemiological work is needed to investigate trends in what is now one of the most common long-term disorders globally. A range of relevant measures of incidence and prevalence, careful use of definitions and description of diagnostic criteria, improved study design, more comprehensive reporting and appropriate interpretation of these data are all essential to ensure that this important field of epidemiological enquiry progresses in a scientifically robust manner.

## Introduction

Atopic eczema is a very common inflammatory skin disorder [1]. Its prevalence appears to vary across the world as noted in key international epidemiological studies [2]–[5]. Such variation has been found in both children and adults and points to the likely importance of environmental risk factors. In addition, atopic eczema has been shown to cluster in families and there is growing evidence that it is an herald condition in many people who go on to develop allergic problems affecting other organ systems (e.g. food allergy) [6], [7]. Genetics are important in the aetiology of atopic eczema: in particular, recent genetic epidemiological studies found a strong association between filaggrin gene defects (present in 1 in 10 Europeans and North Americans), and atopic eczema [7]. Filaggrin plays a role in maintaining the epidermal skin barrier function, whereby it helps to retain moisture in the skin and limits penetration by allergens. These functions can be impaired in filaggrin loss-of-function mutations, this resulting in dry, scaly skin, which increases the risk of allergic sensitisation and disease [7]–[9].

Monitoring disease trends over time aids aetiological understanding and helps with the planning of health services nationally and internationally. Building on our previous work on asthma, we sought to describe international trends in the incidence and prevalence of atopic eczema [10]. We aimed to draw preferentially on high quality studies using appropriate study designs and, in particular, studies using validated instruments [such as the International Study of Asthma and Allergies in Childhood (ISAAC) or the European Community Respiratory Health Survey (ECRHS)] [11], [12].

## Methods

This review is reported using the Preferred Reporting Items for Systematic Reviews and Meta-Analyses (PRISMA) statement as a guide (see Appendix S1) [13]. The methods for this review were specified in advance and documented in a study protocol.

Our full search strategy is given in Appendix S2. In short, we searched seven electronic databases, namely Medline, CINAHL, Embase, Global Health, Global Health Library, Google Scholar and Web of Knowledge, from 1 January 1990 to 19 May 2010 (date of last search). We used both Medical Subject Headings (MeSH) and free text terms of the following concepts: (atopic eczema OR atopic dermatitis) AND (cohort studies OR cross-sectional studies OR ISAAC OR ECRHS) AND (incidence OR prevalence OR trend). The searches were not limited by age, sex, ethnicity or language. Furthermore, bibliographies of key reports were scanned and a citation search was conducted for any additional papers of interest. We only included full-text reports of cohort studies, repeated cross-sectional surveys or analyses of routine healthcare datasets, as we considered these appropriate designs for the assessment of disease trends. Studies were required to present at least two estimates of atopic eczema incidence and/or prevalence within the period 1990 to 2010 and, at each assessment time point, they needed to use a similar approach and instrument (see Table 1). The screening of titles and abstracts and the eligibility assessment of full-text reports was independently performed by two reviewers. Disagreements were resolved by discussion or by a third reviewer if agreement could not be reached. Similarly, to establish the methodological quality of each study, the internal and external validity was examined using the Critical Appraisal Skills Programme (CASP) tool [14] and scored as ‘good’, ‘moderate’ or ‘poor’. This methodological assessment included for example an appraisal of whether validated instruments were used [i.e. at least one of the ISAAC key questions (see Table 2)]. Reviewers were not masked when assessing study quality. Incidence and/or prevalence data as well as study and participant characteristics were extracted onto a customised data extraction sheet by one reviewer and thoroughly checked by the second reviewer.

**Table 1:** Inclusion criteria.

1. Epidemiological design (e.g. cohort, repeated cross-sectional or routine health care)
2. Estimates of eczema incidence and or prevalence at least twice within the period 1990–2010
3. Use of a comparable approach and instrument to measure eczema at each time point.

**Table 2:** Key question for atopic eczema from the ISAAC questionnaire.

Have you ***ever*** had an itchy rash which was coming and going for at least six months?
Have you had this itchy rash at any time ***in the last 12 months?***
Has this itchy rash ***at any time*** affected any of the following places: the folds of the elbows, behind the knees, in front of the ankles, under the buttocks, or around the neck, ears or eyes?
At what age did this itchy rash first occur; under 2 years, age 2-4 years or age 5 or more?
Has this rash cleared completely at any time ***during the last 12 months?***
***In the last 12 months***, how often, on average, have you been kept awake at night by this itchy rash; never in the last 12 months, less than one night per week or one or more nights per week?
Have you ***ever*** had eczema?

To compare disease trends, our primary outcome measure was the lifetime prevalence of symptoms suggestive of atopic eczema or the incidence of atopic eczema (see Table 3). We also collected data on the secondary outcomes, such as the lifetime prevalence of physician-diagnosed eczema or 12-month prevalence measures. There was too much heterogeneity of populations studied and methods employed to undertake meta-analysis.

**Table 3:** Primary and secondary outcomes measures.

**Primary outcomes**	Lifetime prevalence of atopic eczema symptoms
	Incidence of atopic eczema
**Secondary outcomes**	Lifetime prevalence of physician diagnosis of atopic eczema
	12-month prevalence of atopic eczema symptoms
	12-month prevalence of physician diagnosis of atopic eczema

## Results

**Figure 1:**
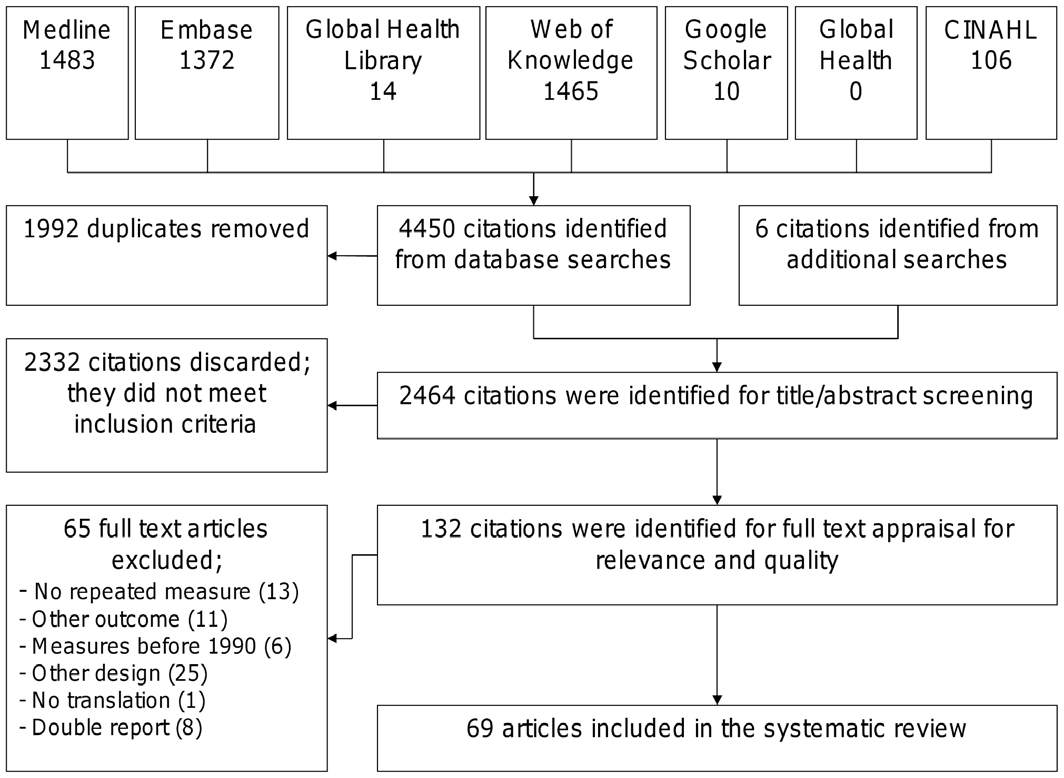
PRISMA flow diagram.

**Figure 2:**
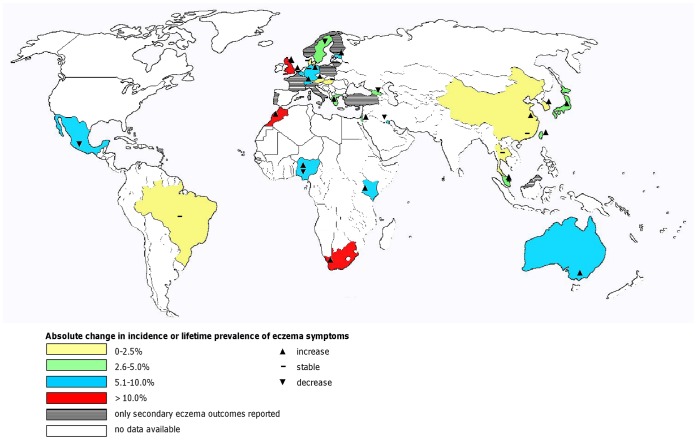
World map of the incidence and lifetime prevalence of atopic eczema symptoms (1990–2010). Overview of absolute changes in the incidence of atopic eczema and lifetime prevalence of atopic eczema symptoms between 1990 and 2010.

Our searches retrieved 2,464 titles from which we identified 70 papers that satisfied our inclusion criteria (see Figure 1). We excluded one of these studies because the full-text paper was only available in Korean [15] and we were unable to procure a translation; there were therefore 69 papers in our final dataset. Data from included studies judged to be of moderate or good quality are summarised in Table 4 and explored descriptively by region (see Tables 5, 6, 7, 8 and 9) [16]. Data from the primary outcomes are additionally represented on a map (see Figure 2). Data from studies judged to be at greater risk of bias are available from the corresponding author [17]–[22]. Nearly all studies had prevalence data, while incidence data were only reported in three European studies [23]–[25]. Prevalence data are described using lifetime prevalence of atopic eczema symptoms.

**Table 4:** Summary of trends in different atopic eczema outcomes between 1990 and 2010 by region*.

Region	Papers	Trends	Incidence			Lifetime prevalence						12-month prevalence					
	(n)	(n)				*symptoms*			*diagnosis*			*symptoms*			*diagnosis*		
			↑	↔	↓	↑	↔	↓	↑	↔	↓	↑	↔	↓	↑	↔	↓
***Africa***	**4**	**20**	0	0	0	9	0	1	2	0	2	4	1	1	0	0	0
***Asia***	**20**	**61**															
eastern	10	27	0	0	0	7	2	0	7	1	1	7	1	0	0	0	1
south-eastern	4	20	0	0	0	2	4	0	1	3	0	4	6	0	0	0	0
western	6	14	0	0	0	1	0	3	0	3	2	0	2	2	0	0	1
***Americas***	**5**	**21**															
North	0	0	0	0	0	0	0	0	0	0	0	0	0	0	0	0	0
Central	1	6	0	0	0	0	0	2	2	0	0	0	0	2	0	0	0
South	4	15	0	0	0	0	5	0	0	1	3	0	5	1	0	0	0
***Europe***	**31**	**101**															
western	10	42	1	1	0	4	2	0	12	5	0	4	9	0	0	0	4
southern	4	15	0	0	0	1	1	0	4	1	0	5	1	0	2	0	0
northern	15	41	1	1	0	9	1	1	15	1	0	8	2	1	0	1	0
eastern	2	3	0	0	0	0	0	0	1	2	0	0	0	0	0	0	0
***Oceania***	**3**	**4**	0	0	0	1	0	0	1	1	0	1	0	0	0	0	0

* Based on UN classification [16].

### Africa

As presented in Table 4, we found four studies on atopic eczema trends for Africa [26]–[29]. Incidence was not measured in any of these studies. Prevalence was measured based on parental- or self-report as assessed by ISAAC-based questions (see Table 5). Data were mainly from 13–14 year old children and in these children the general trend in Africa (Kenya, Morocco and South Africa) for the prevalence of atopic eczema was increasing [25], [27], [28] [26], [27], [29]. In these children, an approximate doubling of the lifetime prevalence of atopic eczema symptoms was found for Morocco [e.g. flexural rash in Marrakech, Morocco – from 9.9% (1995) to 20.9% (2001–02)] [26], for South Africa [e.g. flexural rash – from 10.2% (1995) to 16.5% (2002)] [29] and for Kenya [e.g. itchy recurrent rash in flexural areas – from 11.4% (1995) to 19.8% (2001)] [27]. In Nigeria in children of this age group, the lifetime prevalence of itchy rash decreased from a high baseline prevalence [from 26.1% (1995) to 18.0% (2001–02)] [28]. Prevalence estimates in 2001–02 were, however, comparable for all countries. An approximate doubling was also seen in the lifetime prevalence of physician-diagnosed atopic eczema in 13–14 year olds in South Africa and Kenya [27], [29]. In contrast, the prevalence of physician-diagnosed atopic eczema in Nigeria considerably decreased over a 5-year period from 1995 to 2001 in 6–7 year olds [from 9.4% to 6.8%] and in 13–14 year olds [from 38.4% to 19.4%] [28]. The baseline estimate for 13–14 year olds was again extremely high. In other African countries, single estimates of atopic eczema prevalence may have been reported, but we were unable to locate any serial data on trends.

**Table 5:** Good and moderate quality studies reporting the prevalence of parental- or self-report of atopic eczema between 1990 and 2010 in Africa.

Study	Geographic area	Age range	Outcome	Time period	Baseline estimate		Final estimate		Summary measures	Conclusion	Quality
		(y)			N	% (95%CI)/(SE)**	N	% (95%CI)/(SE)**			
*Measures of symptoms of atopic eczema*											
Falade et al. (2009) [28]	Nigeria (Ibadan)	6–7	ISAAC-based parental-report of:	1995/2001–02	1,696		2,396		% change (S.E.)		Moderate
			lifetime prevalence of itchy rash			7.7 (0.7)		10.2 (0.6)	2.5 (0.9), P = 0.007	Increase	
			12-month prevalence of itchy rash			4.5 (0.5)		5.0 (0.5)	0.5 (0.7), P = 0.437	Stable	
Falade et al. (2009) [28]	Nigeria (Ibadan)	13–14	ISAAC-based parental-report of:	1995/2001–02	3,057		3,142		% change (S.E.)		Moderate
			lifetime prevalence of itchy rash			26.1 (0.8)		18.0 (0.7)	−8.1 (1.0), P<0.001	Decrease	
			12-month prevalence of itchy rash			17.7 (0.7)		7.7 (0.5)	−10.0 (0.8), P<0.001	Decrease	
Bouayad et al. (2006) [26]	Morocco (Casablanca)	13–14	ISAAC-based self-report of:	1995/2001–02	3,178		1,744		% change per year		Moderate
			lifetime prevalence of rash			20.5		34.2 (33.4–35.0)	2.28, P<0.001	Increase	
			lifetime prevalence of flexural rash			12.6		23.9 (23.2–24.6)	1.88, P<0.001	Increase	
			12-month prevalence of rash			14.2		26.1 (25.3–26.8)	1.98, P<0.001	Increase	
Bouayad et al. (2006) [26]	Morocco (Marrakech)	13–14	ISAAC-based self-report of:	1995/2001–02	2,896		1,677		% change per year		Moderate
			lifetime prevalence of rash			20.4		33.9 (33.1–34.7)	2.20, P<0.001	Increase	
			lifetime prevalence of flexural rash			9.9		20.9 (20.2–21.7)	1.79, P<0.001	Increase	
			12-month prevalence of rash			13.1		23.1 (22.3–23.8)	1.63, P<0.001	Increase	
Esamai et al. (2002) [27]	Kenya (Uasin Gishu)	13–14	ISAAC-based self-report of:	1995/2001	3,018		3,258				Moderate
			lifetime prevalence of itchy recurrent rash			23.8		28.5	P = 0.001	Increase	
			lifetime prevalence of itchy recurrent rash in flexural areas			11.4		19.8	P = 0.001	Increase	
			12-month prevalence itchy recurrent rash			14.4		21.3	P = 0.001	Increase	
Zar et al. (2007) [29]	South Africa (Cape Town)	13–14	ISAAC-based self-report of:	1995/2002	5,161		5,019		OR (95%CI)		Moderate
			lifetime prevalence of itchy rash			15.5		26.2	1.93 (1.75–2.14), P<0.001	Increase	
			lifetime prevalence of flexural rash			10.2		16.5	1.75 (1.56–1.97), P<0.001	Increase	
			12-month prevalence itchy rash			11.8		19.4	1.77 (1.56–1.97), P<0.001	Increase	
*Measures of physician-diagnosed atopic eczema*											
Falade et al. (2009) [28]	Nigeria (Ibadan)	6–7	ISAAC-based parental-report of:	1995/2001–02	1,696		2,396		% change (S.E.)		Moderate
			lifetime prevalence of physician-diagnosed atopic eczema			9.4 (0.7)		6.8 (0.5)	−2.6 (0.9), P = 0.003	Decrease	
Falade et al. (2009) [28]	Nigeria (Ibadan)	13–14	ISAAC-based self-report of:	1995/2001–02	3,057		3,142		% change (S.E.)		Moderate
			lifetime prevalence of physician-diagnosed atopic eczema			38.4 (0.9)		19.4 (0.7)	−19.0 (1.1), P<0.001	Decrease	
Esamai et al. (2002) [27]	Kenya (Uasin Gishu)	13–14	ISAAC-based self-report of:	1995/2001	3,018		3,258				Moderate
			lifetime prevalence of atopic eczema			13.9		28.5	P = 0.001	Increase	
Zar et al. (2007) [29]	South Africa (Cape Town)	13–14	ISAAC-based self-report of:	1995/2002	5,161		5,019		OR (95% CI)		Moderate
			lifetime prevalence of physician-diagnosed atopic eczema			9.6		16.7	1.88 (1.67–2.12), P<0.001	Increase	

Abbreviations – CI: confidence intervals, SE: standard error, OR: odds ratio.

* Based on UN classification [16].

** 95% CI and SE are only reported if included in original report.

### Asia

For Asia, we summarised 20 papers representing 61 measures of trends in Table 4. The majority of data came from eastern Asia [30]–[39], whereas south-eastern Asia [40]–[43] and western Asia [44]–[49] were represented to a lesser extent. For other regions in Asia, we found no relevant data. Here too no study assessed trends in incidence; rather, each study measured prevalence as based on parental- or self-report by questionnaires (see Tables 6). Trends were found for different age groups in 12 different countries and showed no overall pattern.

**Table 6:** Good and moderate quality studies reporting the prevalence of parental- or self-report of atopic eczema between 1990 and 2010 in Asia.

Study	Geographic area	Age range	Outcome	Time period	Baseline estimate		Final estimate		Summary measures	Conclusion	Quality
		(y)			N	% (95%CI)/(SE)**	N	% (95%CI)/(SE)**			
Eastern Asia*											
*Measures of symptoms of atopic eczema*											
Liao MF et al. (2009) [34]	Central Taiwan (Changhwa County)	6–8	ISAAC-based parental-report of:	2002/2007	7,040		4,622		POR (95% CI)		Good
			lifetime prevalence of chronic rash			5.8		7.7	1.39 (1.20–1.61), P<0.001	Increase	
			lifetime prevalence of chronic rash with typical distribution			5.9		8.9	1.56 (1.34–1.83), P<0.001	Increase	
			12-month prevalence of chronic rash			7.0		9.7	1.45 (1.25–1.67), P<0.001	Increase	
Liao PF et al. (2009) [35]	Taiwan	6–15	ISAAC-based parental-report of:	1994/2002	75,960		11,580		No formal test		Moderate
			12-month prevalence of current atopic eczema symptoms			1.5		2.8	–	Increase	
Lee et al. (2007) [32]	Taiwan	12–15	ISAAC-based parental-report of:	1995–96/2001	42,919		10,215		Adjusted PR (95% CI)		Good
			Sex- and age-standardised lifetime prevalence of atopic eczema symptoms			2.4		4.0	1.61 (1.42–1.81), P<0.001	Increase	
Yan et al. (2005) [38]	Taiwan (Taipei)	13–14	ISAAC-based self-report of:	1994–95/2001–02	11,400		6,303				Moderate
			12-month prevalence of recurrent itchy rash in a typical distribution			1.4 (1.1–1.6)		4.1 (3.6–4.6)	P<0.001	Increase	
Lee et al. (2004) [31]	China (Hong Kong)	6–7	ISAAC-based parental-report of:	1995/2001	3,618		4,448		OR (95% CI)		Moderate
			lifetime prevalence of chronic rash			5.7		5.4	0.95 (0.79–1.15), P = 0.56	Stable	
			lifetime prevalence of chronic rash at typical areas			4.2		3.6	0.85 (0.68–1.07), P = 0.18	Stable	
			12-month prevalence of chronic rash			4.2		4.2	1.00 (0.80–1.25), P = 1.00	Stable	
Wang et al. (2006) [37]	China (Guangzhou city)	13–14	ISAAC-based self-report of:	1994–95/2001	3,855		3,516				Moderate
			lifetime prevalence of flexural atopic eczema symptoms			1.7 (1.3–2.1)		3.0 (2.4–3.6)	P<0.05	Increase	
			12-month prevalence of flexural atopic eczema symptoms			1.3 (0.9–1.7)		2.2 (1.7–2.7)	P = 0.002	Increase	
Oh et al. (2004) [36]	Korea	6–12	ISAAC-based parental-report of:	1995/2000	25,361		27,425		No formal test		Moderate
			lifetime prevalence of itchy atopic eczema symptoms			15.3 (14.9–15.8)		17.0 (16.5–17.4)		Increase	
			12-month prevalence of itchy flexural atopic eczema symptoms			7.3 (7.0–7.6)		10.7 (10.4–11.1)		Increase	
Oh et al. (2004) [36]	Korea	12–15	ISAAC-based parental-report of:	1995/2000	15,068		14,777		No formal test		Moderate
			lifetime prevalence of itchy atopic eczema symptoms			7.2 (6.8–7.7)		9.3 (8.8–9.8)		Increase	
			12-month prevalence of itchy flexural atopic eczema symptoms			3.9 (3.6–4.3)		6.1 (5.7–6.5)		Increase	
Kusunoki et al. (2009) [30]	Japan (Kyoto)	7–15	Parental-report of:	1996/2006	16,176		13,215				Moderate
			lifetime prevalence of symptoms of atopic dermatitis			10.1		13.6	P<0.0001	Increase	
			12-month prevalence of symptoms of atopic dermatitis			4.2		5.6	P<0.0001	Increase	
*Measures of physician-diagnosed atopic eczema*											
Liao MF et al. (2009) [34]	Taiwan (Changhwa County)	6–8	ISAAC-based parental-report of:	2002/2007	7,040		4,622		POR (95% CI)		Good
			lifetime prevalence of physician-diagnosed atopic eczema			18.0		23.9	1.44 (1.31–1.57), P<0.001	Increase	
Liao PF et al. (2009) [35]	Taiwan	6–15	ISAAC-based parental-report of:	1994/2002	75,960		11,580		No formal test		Moderate
			lifetime prevalence of atopic eczema			1.9		3.4		Increase	
Lee et al. (2005) [33]	Taiwan	12–15	ISAAC-based parental-report of:	1995–96/2001	44,104		11,048		No formal test		Moderate
			lifetime prevalence of physician-diagnosed atopic eczema			1.6		2.8		Increase	
Yan et al. (2005) [38]	Taiwan (Taipei)	13–14	ISAAC-based self-report of:	1994–95/2001–02	11,400		6,303				Moderate
			lifetime prevalence of atopic eczema			11.8 (11.2–12.4)		17.4 (16.4–18.3)	P<0.001	Increase	
Lee et al. (2004) [31]	China (Hong Kong)	6–7	ISAAC-based parental-report of:	1995/2001	3,618		4,448		OR (95% CI)		Moderate
			lifetime prevalence of atopic eczema			28.1		30.7	1.13 (1.03–1.25), P = 0.01	Increase	
Wang et al. (2006) [37]	China (Guangzhou city)	13–14	ISAAC-based self-report of:	1994–95/2001	3,855		3,516				Moderate
			lifetime prevalence of physician-diagnosed atopic eczema			18.3 (17.1–19.5)		17.6 (16.3–18.9)	P = 0.462	Stable	
Oh et al. (2004) [36]	Korea	6–12	ISAAC-based parental-report of:	1995/2000	25,361		27,425		No formal test		Moderate
			lifetime prevalence of physician-diagnosed atopic eczema			16.6 (16.2–17.1)		24.9 (24.4–25.4)		Increase	
Oh et al. (2004) [36]	Korea	12–15	ISAAC-based parental-report of:	1995/2000	15,068		14,777		No formal test		Moderate
			lifetime prevalence of physician-diagnosed atopic eczema			7.3 (6.9–7.7)		12.8 (12.3–13.3)		Increase	
Yura et al. (2001) [39]	Japan (Osake Prefecture)	7–12	Parental-report of:	1993/1997	514,656		458,284		No formal test		Moderate
			lifetime prevalence of physician-diagnosed atopic dermatitis			24.1		22.9		Decrease	
			12-month prevalence of physician-diagnosed atopic dermatitis			6.8		5.7		Decrease	
**South-eastern Asia** *											
*Measures of symptoms of atopic eczema*											
Wang et al. (2004) [43]	Singapore	6–7	ISAAC-based parental-report of:	1994/2001	2,030		5,305		% Change (S.E.)		Good
			lifetime prevalence of chronic rash			10.5 (1.2)		12.5 (0.5)	2.0 (1.3), P = 0.194	Stable	
			lifetime prevalence of chronic rash with typical distribution			6.1 (0.9)		9.8 (0.4)	3.7 (1.0), P = 0.028	Increase	
			12-month prevalence of chronic rash			8.9 (1.1)		11.0 (0.4)	2.1 (1.2), P = 0.155	Stable	
Wang et al. (2004) [43]	Singapore	12–15	ISAAC-based parental-report of:	1994/2001	4,208		4,058		% Change (S.E.)		Good
			lifetime prevalence of chronic rash			12.3 (0.5)		14.9 (0.6)	2.6 (0.8), P = 0.056	Stable	
			lifetime prevalence of chronic rash with typical distribution			7.0 (0.4)		10.2 (0.5)	3.2 (0.6), P<0.001	Increase	
			12-month prevalence of chronic rash			9.5 (0.5)		11.6 (0.5)	2.1 (0.7), P = 0.034	Increase	
Quah et al. (2005) [40]	Malaysia (Kota Bharu)	6–7	ISAAC-based parental-report of:	1995/2001	3,939		3,157		% Change (95% CI)		Good
			12-month prevalence of flexural itchy rash			14.0		17.6	3.6 (1.3–5.9), P = 0.004	Increase	
Quah et al. (2005) [40]	Malaysia (Kota Bharu)	13–14	ISAAC-based parental-report of:	1995/2001	3,116		3,004		% Change (95% CI)		Good
			12-month prevalence of flexural itchy rash			12.1		13.4	1.3 (–4.6–7.1), P = 0.11	Stable	
Trakultivakorn et al. (2007) [42]	Thailand (Chiang Mai)	6–7	ISAAC-based parental-report of:	1995/2001	3,828		3,106				Moderate
			12-month prevalence of atopic eczema symptoms			11.4		16.3	P<0.01	Increase	
Trakultivakorn et al. (2007) [42]	Thailand (Bangkok)	6–7	ISAAC-based parental-report of:	1995/2001	3,628		3,430				Moderate
			12-month prevalence of atopic eczema symptoms			12.5		13.3	P = 0.33	Stable	
Teeratakulpisarn et al. (2004) [41]	Thailand (Northeast)	6–7	parental-report of:	1998–99/2003	2,658		2,119		No formal test		Moderate
			lifetime prevalence of rash			18.0		17.2		Stable	
			12-month prevalence of rash			15.2		14.7		Stable	
Trakultivakorn et al. (2007) [41]	Thailand (Chiang Mai)	13–14	ISAAC-based parental-report of:	1995/2001	3,927		3,538				Moderate
			12-month prevalence of atopic eczema symptoms			9.6		8.6	P = 0.63	Stable	
Trakultivakorn et al. (2007) [41]	Thailand (Bangkok)	13–14	ISAAC-based parental-report of:	1995/2001	3,713		4,669				Moderate
			12-month prevalence of atopic eczema symptoms			6.8		10.4	P<0.01	Increase	
Teeratakulpisarn et al. (2004) [41]	Thailand (Northeast)	13–14	parental-report of:	1998–99/2003	3,410		2,956		No formal test		Moderate
			lifetime prevalence of rash			9.9		10.9		Stable	
			12-month prevalence of rash			7.4		8.7		Stable	
*Measures of physician-diagnosed atopic eczema*											
Wang et al. (2004) [43]	Singapore	6–7	ISAAC-based parental-report of:	1994/2001	2,030		5,305		% Change (S.E.)		Good
			lifetime prevalence of physician-diagnosed atopic eczema			3.0 (0.7)		8.8 (0.4)	5.8 (0.8), P<0.001	Increase	
Wang et al. (2004) [43]	Singapore	12–15	ISAAC-based parental-report of:	1994/2001	4,208		4,058		% Change (S.E.)		Good
			lifetime prevalence of physician-diagnosed atopic eczema			4.1 (0.3)		5.8 (0.4)	1.7 (0.5), P = 0.810	Stable	
Teeratakulpisarn et al. (2004) [41]	Thailand (Northeast)	6–7	Parental-report of:	1998–99/2003	2,658		2,119		No formal test		Moderate
			lifetime prevalence of atopic eczema			30.5		29.2		Stable	
Teeratakulpisarn et al. (2004) [41]	Thailand (Northeast)	13–14	Self-report of:	1998–99/2003	3,410		2,956		No formal test		Moderate
			lifetime prevalence of atopic eczema			24.4		26.8		Stable	
**Western Asia** *											
*Measures of symptoms of atopic eczema*											
Abramidze et al. (2006) [44]	Georgia (Tbilisi)	6–7	ISAAC-based parental-report of:	1996/2003	6,770		6,002		% Change		Moderate
			lifetime prevalence of symptoms of flexural dermatitis			4.5		3.4	−1.1, P<0.05	Decrease	
			current prevalence of itchy rash			5.3		5.8	0.5, P = not significant	Stable	
Abramidze et al. (2006) [44]	Georgia (Kutaisi)	6–7	ISAAC-based parental-report of:	1996/2003					% Change		Moderate
			lifetime prevalence of symptoms of flexural dermatitis			5.2		2.4	−2.8, P<0.05	Decrease	
			current prevalence of itchy rash			6.1		3.4	−2.7, P<0.05	Decrease	
Abramidze et al. (2007) [45]	Georgia (Tbilisi and Kutaisi)	13–14	ISAAC-based self-report of:	1996/2003	6,746		5,653		% Change		Moderate
			current prevalence of itchy rash			4.1		4.3	0.2, P = not significant	Stable	
Owayed et al. (2008) [48]	Kuwait	13–14	ISAAC-based self-report of:	1995–96/2001–02	3,110		2,822				Moderate
			lifetime prevalence itchy rash			17.5 (16.2–18.8)		10.6 (9.5–11.7)	P<0.001	Decrease	
			12-month prevalence of itchy rash			12.6 (11.4–13.8)		8.3 (7.3–9.3)	P<0.001	Decrease	
Romano-Zelekha et al. (2007) [49]	Israel	13–14	ISAAC-based self-report of:	1997/2003	10,057		8,978				Moderate
			lifetime prevalence of itchy rash in a typical distribution			5.9		8.7	P<0.05	Increase	
*Measures of physician-diagnosed atopic eczema*											
Abramidze et al. (2006) [44]	Georgia (Tbilisi)	6–7	ISAAC-based parental-report of:	1996/2003	6,770		6,002		% Change		Moderate
			lifetime prevalence of physician-diagnosed atopic eczema			11.6		3.6	−8, P<0.05	Decrease	
Abramidze et al. (2006) [44]	Georgia (Kutaisi)	6–7	ISAAC-based parental-report of:	1996/2003					% Change		Moderate
			lifetime prevalence of physician-diagnosed atopic eczema			4.7		1.8	−2.9, P<0.05	Decrease	
Abramidze et al. (2007) [45]	Georgia (Tbilisi and Kutaisi)	13–14	ISAAC-based self-report of:	1996/2003	6,746		5,653		% Change		Moderate
			lifetime prevalence of physician-diagnosed atopic eczema			3.0		2.6	−0.4, P = not significant	Stable	
Owayed et al. (2008) [48]	Kuwait	13–14	ISAAC-based self-report of:	1995–96/2001–02	3,110		2,822		P = 0.101		Moderate
			lifetime prevalence of physician-diagnosed atopic eczema			11.3 (10.2–12.4)		12.8 (11.6–14)		Stable	
Kalyoncu et al. (1999) [47]	Turkey (Ankara)	6–13	ISAAC-based self-report of:	1992/1997	1,036		738		P = not significant		Moderate
			lifetime prevalence of physician-diagnosed atopic dermatitis			6.1 (4.7–7.7)		6.5 (4.8–8.5)		Stable	
Demir et al. (2010) [46]	Turkey (Ankara)	7–12	Parental-report of:	1992/2007	1,036		442		Adjusted POR		Moderate
			current prevalence of atopic eczema			4.0 (2.8–5.2)		1.2 (0.2–2.2)	0.4 (0.2–1.0), P trend = 0.004	Decrease	

Abbreviations – CI: confidence intervals, SE: standard error, OR: odds ratio, POR: prevalence odds ratio, PR: prevalence ratio.

* Based on UN classification [16].

** 95% CI or SE are only reported if included in original report.

# Point estimate extracted from graph or chart.

In eastern Asia, the general trend for atopic eczema prevalence was mainly increasing across different age groups. Lee et al. (2007) reported an increase in the sex- and age-standardised lifetime prevalence of ISAAC-based parental-report of atopic eczema symptoms in Taiwan among 12–15 year olds [from 2.4% (1995–96) to 4.0% (2001)] [32]. The lifetime prevalence of atopic eczema symptoms also increased in Korea in the same age group [from 7.2% (1995) to 9.3% (2000)] [36], in China (Guangzhou city) in a similar age group 13–14 [from 1.7% (1994–95) to 3.0% (2001)] [37] and in Japan in a wider-ranged age group 7–15 [from 10.1% (1996) to 13.6% (2006)] [39]. Moreover, baseline prevalences were low, but considerably higher in Korea and Japan, compared to Taiwan and China. In a slightly younger age group 6–12 in Korea, atopic eczema symptoms showed a modest increase from a substantially higher baseline prevalence [from 15.3% (1995) to 17.0% (2000)] [36]. In the youngest children aged 6–7, the prevalence of atopic eczema symptoms was stable in Hong Kong [e.g. chronic rash – from 5.7% (1995) to 5.4% (2001)] [31], whilst a modest increase was seen in a later study in Taiwan in a similar age group 6–8 [e.g. chronic rash - from 5.8% (2002) to 7.7% (2007)] [34]. Trends in the lifetime prevalence of physician-diagnosed atopic eczema followed nearly the same pattern as the lifetime prevalence of atopic eczema symptoms; trends were increasing in most countries among different age groups with only few exceptions.

In south-eastern Asia, the prevalence of different atopic eczema symptoms showed mixed trends. For chronic rash, the lifetime prevalence was stable in 6–7 year olds in Singapore [10.5% (1994) and 12.5% (2001)] [42] and in north-eastern Thailand [18.0% (1998–99) and 17.2% (2003)] [41]. Moreover, this prevalence also remained stable in older children (aged 12–15) in Singapore and, even though the baseline prevalence was appreciably lower, in 13–14 year olds in north-eastern Thailand. For chronic rash with a typical distribution, however, the lifetime prevalence was increasing in Singapore in children of both age groups [e.g. in 6–7 year olds – from 6.1% (1994) to 9.8% (2001)] [43]. In Malaysia and two specific geographical areas in Thailand (Chiang Mai and Bangkok) only data regarding the 12-month prevalence of atopic eczema symptoms were available [40], [41]. In Malaysia and Chiang Mai, the 12-month prevalence of atopic eczema symptoms increased in 6–7 year olds, but was stable in 13–14 year olds, whereas the opposite was seen in Bangkok.

In western Asia, data were found for Georgia, Kuwait, Turkey and Israel [44]–[49]. In Georgia, the lifetime prevalence of atopic eczema symptoms was found to be decreasing in two different geographical areas among 6–7 year olds: in Tbilisi [from 4.5% (1996) to 3.4% (2003)] and in Kutaisi [from 5.2% (1996) to 2.4% (2003)] [44]. This trend was additionally apparent in the lifetime prevalence of physician-diagnosed atopic eczema in these children. There was also a decrease in the prevalence of atopic eczema symptoms in 13–14 year old children from Kuwait [from 17.5% (1995–96) to 10.6% (2001–02)], but the baseline prevalence was much higher [48]. In Israel, the lifetime prevalence of itchy rash in a distribution suggestive of atopic eczema was found to be increasing [from 5.9% (1997) to 8.7% (2003)] [49]. In Turkey, two measures of the prevalence of physician-diagnosed atopic eczema were reported. The lifetime prevalence was stable in 6–13 year old children [6.1% (1992) and 6.5% (2007)] [47], whilst the 12-month prevalence was reported to have decreased over a 15-year period in 7–12 year old children [from 4.0% (1992) to 1.2% (2007)] [46].

### The Americas

We found no studies on atopic eczema trends for North America, one study for Central America [50] and four studies for South America [51]–[54] (see Table 4). No studies reported an incidence trend. The study from Central America, which was conducted in Mexico in 6–8 and 11–14 year old children (see Table 7) [50]. This study showed a sharply decreasing lifetime (and 12-month) prevalence of itchy rash in both age groups [e.g. in 6–8 year olds – from 15.0% (1995) to 7.3% (2002)] and, conversely, a from low baseline increasing lifetime prevalence of physician-diagnosed atopic eczema in both age groups [e.g. in 6–8 year olds – from 3.9% (1995) to 6.1% (2002)].

**Table 7:** Good and moderate quality studies reporting the prevalence of parental- or self-report of atopic eczema between 1990 and 2010 in the Americas.

Study	Geographic area	Age range	Outcome	Time period	Baseline estimate		Final estimate		Summary measures	Conclusion	Quality
		(y)			N	% (95%CI)/(SE)**	N	% (95%CI)/(SE)**			
**Central America** *											
*Measures of symptoms of atopic eczema*											
Barraza-villareal et al. (2007) [50]	Mexico (Cuernavaca)	6–8	ISAAC-based parental-report of:	1995/2002	2,770		2,633				Good
			lifetime prevalence of dry itchy skin spots			15.0 (13.8–16.4)		7.3 (6.3–8.4)	P = 0.000	Decrease	
			12-month prevalence of dry itchy skin spots			10.1 (9.1–11.3)		5.8 (4.9–6.8)	P = 0.000	Decrease	
Barraza-villareal et al. (2007) [50]	Mexico (Cuernavaca)	11–14	ISAAC-based parental-report of:	1995/2002	2,795		2,605				Good
			lifetime prevalence of dry itchy skin spots			17.0 (15.6–18.4)		7.0 (6.0–8.1)	P = 0.000	Decrease	
			12-month prevalence of dry itchy skin spots			10.5 (9.5–11.7)		5.4 (4.5–6.3)	P = 0.000	Decrease	
*Measures of physician-diagnosed atopic eczema*											
Barraza-villareal et al. (2007) [50]	Mexico (Cuernavaca)	6–8	ISAAC-based parental-report of:	1995/2002	2,770		2,633				Good
			lifetime prevalence of physician-diagnosed atopic eczema			3.9 (3.2–4.7)		6.1 (5.2–7.2)	P = 0.000	Increase	
Barraza-villareal et al. (2007) [50]	Mexico (Cuernavaca)	11–14	ISAAC-based parental-report of:	1995/2002	2,795		2,605				Good
			lifetime prevalence of physician-diagnosed atopic eczema			4.2 (3.5–5.0)		6.9 (6.0–8.0)	P = 0.000	Increase	
**South America** *											
*Measures of symptoms of atopic eczema*											
Camelo-Nunes et al. (2004) [52]	Brazil (São Paulo)	6–7	ISAAC-based parental-report of:	1996/1999	3,005		3,033				Moderate
			lifetime prevalence of itchy rash			13.6		15.0	P = not significant	Stable	
			lifetime prevalence of lesions in skin-folds			7.5		6.6	P = not significant	Stable	
			12-month prevalence of itchy rash			10.6		9.9	P = not significant	Stable	
Camelo-Nunes et al. (2004) [52]	Brazil (São Paulo)	13–14	ISAAC-based self-report of:	1996/1999	3,008		3,487				Moderate
			lifetime prevalence of itchy rash			12.6		14.0	P = not significant	Stable	
			lifetime prevalence of lesions in skin-folds			4.8		4.6	P = not significant	Stable	
			12-month prevalence of itchy rash			8.1		8.8	P = not significant	Stable	
Borges et al. (2008) [51]	Brazil (Federal district of Brasilia)	13–14	ISAAC-based self-report of:	1996/2002	3,254		3,009				Moderate
			lifetime prevalence of itchy rash			15.5		16.8	P = 0.185	Stable	
			12-month prevalence of itchy rash			9.2		10.2	P = 0.202	Stable	
Solé et al (2007) [54]	Brazil (5 centres)	13–14	ISAAC-based self-report of:	1994–95/2001–03	15,419		15,684		OR (95% CI)		Moderate
			12-month prevalence of itchy rash			10.3		8.4	0.80 (0.74–0.86), P<0.05	Decrease, not uniform among centres	
Riedi et al. (2005) [53]	Brazil (Curitiba)	13–14	ISAAC-based self-report of:	1995/2001	3,008		3,628				Moderate
			12-month prevalence of Itchy rash			6.3		6.0	P = not significant	Stable	
			12-month prevalence of Intermittent itchy rash in skin creases			3.7		3.7	P = not significant	Stable	
*Measures of physician-diagnosed atopic eczema*											
Camelo-Nunes et al. (2004) [52]	Brazil (São Paulo)	6–7	ISAAC-based parental-report of:	1996/1999	3,005		3,033				Moderate
			lifetime prevalence of physician-diagnosed atopic eczema			13.2		11.4	P<0.05	Decrease	
Camelo-Nunes et al. (2004) [52]	Brazil (São Paulo)	13–14	ISAAC-based parental-report of:	1996/1999	3,008		3,487				Moderate
			lifetime prevalence of physician-diagnosed atopic eczema			14.0		15.0	P = not significant	Stable	
Borges et al. (2008) [51]	Brazil (Federal district of Brasilia)	13–14	ISAAC-based self-report of:	1996/2002	3,254		3,009				Moderate
			lifetime prevalence of physician-diagnosed atopic eczema			9.8		13.6	P = 0.0002	Decrease	
Solé et al (2007) [54]	Brazil (5 centres)	13–14	ISAAC-based self-report of:	1994–95/2001–03	15,419		15,684		OR (95% CI)		Moderate
			lifetime prevalence of physician-diagnosed atopic eczema			5.3		4.5	0.84 (0.76–0.93), P<0.05	Decrease, not uniform among centres	

Abbreviations – CI: confidence intervals, SE: standard error, OR: odds ratio.

* Based on UN classification [16].

** 95% CI and SE are only reported if included in original report.

All four studies from South America were from Brazil and each study included only trends in prevalence as based on parental- or self-report by questionnaires (see Table 7) [51]–[54]. Two studies measured the lifetime prevalence of atopic eczema symptoms and showed a stable trend among 6–7 and 13–14 year olds [e.g. itchy rash in São Paulo in 6–7 year olds - 13.6% (1996) and 15.0% (1999)] [51], [52]. In another study, the 12-month prevalence of itchy rash remained stable in children aged 13–14 years old [6.3% (1995) and 6.0% (2001)] [53], whereas in the last study both the lifetime prevalence of physician-diagnosed atopic eczema and the 12-month prevalence of atopic eczema symptoms were decreasing in 13–14 year olds across five centres [e.g. physician-diagnosed atopic eczema - from 5.3% (1994–95) to 4.5% (2001–03)] [54].

### Europe

The largest set of reports (n = 31) on atopic eczema trends is for Europe. The majority of all trends were increasing, although decreasing and stable trends were found in some areas (see Table 4).

#### Incidence

Three studies reported on incidence trends in atopic eczema in Europe [23]–[25]. In Denmark, the adjusted cumulative incidence of the UK Working Party-based parental-report of physician-diagnosed atopic eczema in 7 year olds was 18.9% in 1993 and 19.6% in 1998 (see Table 8). Compared to the survey of 1993 the sample size was over nine times larger in the survey of 1998 [23]. Further, the cumulative incidence of parental-report of history of physician-diagnosed atopic eczema in 5–6 year olds was stable in West Germany [12.5% (1991) and 12.8% (1997)], whilst it increased sharply in East Germany [from 9.6% (1991) to 23.4% (1997)] [24]. Finally, the age- and sex-standardised incidence of physicians’ recorded atopic eczema diagnosis as based on secondary analysis of QRESEARCH, a large primary care dataset (n = 333,294) in England, increased from 9.6% (2001) to 13.6% (2005) per 1000 patient-years [25].

**Table 8:** Good and moderate quality studies reporting the incidence of parental- or self-report of atopic eczema between 1990 and 2010 in Europe.

Study	Geographic area	Age range	Outcome	Time period	Baseline estimate		Final estimate		Summary measures	Conclusion	Quality
		(y)			N	% (95%CI)/(SE)**	N	% (95%CI)/(SE)**			
Schäfer et al. (2000) [24]	Germany (west)	5–6	Parental-report of:	1991/1997	4,001		4,001		No formal test		Moderate
			cumulative incidence of history of physician-diagnosed atopic eczema			12.5		12.8		Stable	
Schäfer et al. (2000) [24]	Germany (east)	5–6	Parental-report of:	1991/1997					No formal test		Moderate
			cumulative incidence of history of physician-diagnosed atopic eczema			16.0		23.4		Increase	
Olesen et al. (2005) [23]	Denmark	7	UK working party-based parental-report of:	1993/1998	1,060		9,744				Moderate
			adjusted cumulative incidence of physician-diagnosed atopic dermatitis			18.9		19.6	No formal test	Stable	
Simpson et al. (2009) [25]	UK	all	QRESEARCH-based physicians’ recorded:	2001–05	>30 million py		>30 million py		Relative % Change		Moderate
			age- and sex-standardised incidence of atopic eczema diagnosis (per 1000 patient years (py))			9.6 (9.5–9.7)		13.6 (13.5–13.7)	41.8, P<0.001	Increase	

Abbreviations – CI: confidence intervals, SE: standard error.

* Based on UN classification [16].

** 95% CI and SE are only reported if included in original report.

#### Prevalence

Prevalence data on trends in atopic eczema for western Europe are shown in Table 9 [24], [55]–[63]. Parental- and self-report of atopic eczema symptoms were reported in five countries [55], [56], [59], [60], [62], [63]. The lifetime prevalence of atopic eczema symptoms increased in 5–7 year old children in Switzerland [from 11.7% (1992) to 17.4% (2001)] [56] and in slightly older children (aged 6–9) in Austria, but from a lower baseline [from 9.2% (1995–97) to 11.0% (2001–03)] [63]. This lifetime prevalence of atopic eczema symptoms also increased in Belgium, both in boys and girls aged 6–7 [e.g. in boys - from 12.9% (1995–96) to 18.4% (2002)], whilst in 13–14 year old boys and girls it remained stable [e.g. in boys −15.7% (1995–96) and 13.3% (2002)] [62]. We found no data on the lifetime prevalence of atopic eczema symptoms for Germany and France. In France, the lifetime prevalence of physician-diagnosed atopic eczema increased in 13–14 year olds [from 25.8% (1995) to 30.4% (2002)] [55]. In Germany (Münster), this prevalence also increased in 13–14 year olds [e.g. in boys - from 8.2% (1994–95) to 10.9% (1999–2000)], whilst it showed a stable trend in 6–7 year olds [e.g. in boys −14.3% (1994–95) and 13.6% (1999–2000)] [60].

**Table 9:** Good and moderate quality studies reporting the incidence and prevalence of parental- or self-report of atopic eczema between 1990 and 2010 in Europe.

Study	Geographic area	Age range	Outcome	Time period	Baseline estimate		Final estimate		Summary measures	Conclusion	Quality
		(y)			N	% (95%CI)/(SE)**	N	% (95%CI)/(SE)**			
**Western Europe** *											
*Measures of symptoms of atopic eczema*											
Grize et al. (2006) [56]	Switzerland	5–7	ISAAC-based parental-report of:	1992/2001	988		1,274				Good
			adjusted lifetime prevalence of skin rash			11.7 (9.7–14.0)		17.4 (15.3–19.7)	P = 0.0014	Increase	
			adjusted 12-month prevalence of atopic eczema specific skin rash			4.6 (3.4–6.2)		7.6 (6.2–9.2)	P = 0.0090	Increase	
Vellinga et al. (2005) [62]	Belgium (Antwerp)	6–7	ISAAC-based parental-report of:	1995–96/2002					POR (95% CI)		Good
			lifetime prevalence of rash in boys		2,313	12.9	2,225	18.4	1.5 (1.3–1.8), P = 0.00	Increase	
			lifetime prevalence of rash in girls		2,359	15.7	2,196	19.8	1.3 (1.1–1.5), P = 0.00	Increase	
			12-month prevalence of rash in boys		2,313	8.5	2,225	11.4	1.4 (1.1–1.7), P = 0.00	Increase	
			12-month prevalence of rash in girls		2,359	11.9	2,196	14.7	1.3 (1.1–1.5), P = 0.01	Increase	
Vellinga et al. (2005) [62]	Belgium (Antwerp)	13–14	ISAAC-based parental-report of:	1995–96/2002					POR (95% CI)		Good
			lifetime prevalence of rash in boys		1,240	15.7	1,215	13.3	0.9 (0.7–1.1), P = 0.17	Stable	
			lifetime prevalence of rash in girls		1,150	19.0	1,318	20.3	1.1 (0.9–1.3), P = 0.30	Stable	
			12-month prevalence of rash in boys		1,240	9.7	1,215	8.5	0.9 (0.7–1.1), P = 0.30	Stable	
			12-month prevalence of rash in girls		1,150	13.3	1,318	13.6	1.0 (0.8–1.3), P = 0.84	Stable	
Krämer et al. (2009) [59]	Germany (west)	6	ISAAC-based parental-report of:	1994–95/1996–2000	4,761		3,654		Area-adjusted trend		Good
			12-month prevalence of itchy skin rash			4.6		4.5	0.89 (0.41–1.92)	Stable	
Krämer et al. (2009) [59]	Germany (east)	6	ISAAC-based parental-report of:	1994–95/1996–2000	114,457		9,031		Area-adjusted trend		Good
			12-month prevalence of itchy skin rash			6.3		6.2	0.96 (0.66–1.39)	Stable	
Maziak et al. (2003) [60]	Germany (Münster)	6–7	ISAAC-based parental-report of:	1994–95/1999–2000					POR (95% CI)		Good
			12-month prevalence of atopic eczema symptoms in boys		1,754	7.3	1,863	6.6	0.9 (0.69–1.17)	Stable	
			12-month prevalence of atopic eczema symptoms in girls		1,713	6.7	1,666	9.8	1.5 (1.18–1.97)	Increase	
Maziak et al. (2003) [60]	Germany (Münster)	13–14	ISAAC-based self-report of:	1994–95/1999–2000					POR (95% CI)		Good
			12-month prevalence of atopic eczema symptoms in boys		1,865	5.0	1,894	4.5	0.9 (0.66–1.22)	Stable	
			12-month prevalence of atopic eczema symptoms in girls		1,892	9.4	1,922	11.1	1.2 (0.98–1.50)	Stable	
Weber et al. (2010) [63]	Austria (Upper)	6–9	ISAAC-based parental-report of:	1995–97/2001–03	12,115		11,468		No formal test		Moderate
			lifetime prevalence of rash			9.2		11.0		Increase	
			12-month prevalence of rash			6.0		6.7		Stable	
Annesi-Maesano et al. (2009) [55]	France (Languedoc Roussillon)	13–14	ISAAC-based self-report of:	1995/2002	3,383		1,642		Absolute/relative % Change		Moderate
			12-month prevalence of atopic eczema symptoms			12.5		14.3	1.78/0.14, P = not significant	Stable	
*Measures of physician-diagnosed atopic eczema*											
Grize et al. (2006) [56]	Switzerland	5–7	ISAAC-based parental-report of:	1992/2001	988		1,274				Good
			adjusted lifetime prevalence of physician-diagnosed atopic eczema			18.4 (15.8–21.2)		15.2 (13.2–17.4)	P trend = 0.1065	Stable	
Vellinga et al. (2005) [62]	Belgium (Antwerp)	6–7	ISAAC-based parental-report of:	1995–96/2002					POR (95% CI)		Good
			lifetime prevalence atopic eczema in boys		2,313	18.5	2,225	20.8	1.2(1.0–1.3), P = 0.06	Increase	
			lifetime prevalence atopic eczema in girls		2,359	19.1	2,196	22.4	1.2(1.1–1.4), P = 0.01	Increase	
Vellinga et al. (2005) [62]	Belgium (Antwerp)	13–14	ISAAC-based parental-report of:	1995–96/2002					POR (95% CI)		Good
			lifetime prevalence atopic eczema in boys		1,240	23.4	1,215	21.1	0.9(0.7–1.1), P = 0.17	Stable	
			lifetime prevalence atopic eczema in girls		1,150	27.8	1,318	29.7	1.1(0.9–1.3), P = 0.30	Stable	
Schäfer et al. (2000) [24]	Germany (west)	5–6	Report of:	1991/1997	801		771		No formal test		Moderate
			current prevalence of physician-diagnosed atopic eczema			11.2		4.5		Decrease	
Schäfer et al. (2000) [24]	Germany (east)	5–6	Report of:	1991/1997	285		633		No formal test		Moderate
			current prevalence of physician-diagnosed atopic eczema			17.5		11.2		Decrease	
Krämer et al. (2009) [59]	Germany (west)	6	Report of:	1991–95/1996–2000	4,761		3,654		Area-adjusted trend (10 y)		Good
			current prevalence of physician-diagnosed atopic eczema			10.5		5.2	0.30 (0.17–0.53)	Decrease	
Krämer et al. (2009) [59]	Germany (east)	6	Report of:	1991–1995/1996–2000	114,457		9,031		Area-adjusted trend (10 y)		Good
			current prevalence of physician-diagnosed atopic eczema			14.3		10.5	0.36 (0.17–0.61)	Decrease	
Maziak et al. (2003) [60]	Germany (Münster)	6–7	ISAAC-based parental-report of:	1994–95/1999–2000					POR (95% CI)		Good
			lifetime prevalence of physician-diagnosed atopic eczema in boys		1,754	14.3	1,863	13.6	0.9 (0.77–1.13)	Stable	
			lifetime prevalence of physician-diagnosed atopic eczema in girls		1,713	14.6	1,666	16.9	1.2 (0.99–1.44)	Stable	
Maziak et al. (2003) [60]	Germany (Münster)	13–14	ISAAC-based parental-report of:	1994–95/1999–2000					POR (95%CI)		Good
			lifetime prevalence of physician-diagnosed atopic eczema in boys		1,865	8.2	1,894	10.9	1.4 (1.09–1.71)	Increase	
			lifetime prevalence of physician-diagnosed atopic eczema in girls		1,892	12.3	1,922	17.4	1.5 (1.22–1.77)	Increase	
Heinrich et al. (2002) [58]	Germany (east)		Parental-report of:	1992–1993/1998–1999	2,773		3,092		No formal test		Moderate
		6	adjusted lifetime prevalence of physician-diagnosed atopic eczema			8.6		13.0		Increase	
		9	adjusted lifetime prevalence of physician-diagnosed atopic eczema			8.6		11.8		Increase	
		12	adjusted lifetime prevalence of physician-diagnosed atopic eczema			9.6		10.2		Increase	
Schernhammer et al. (2008) [61]	Austria (Upper)	6–7	ISAAC-based parental-report of:	1995–97/2001–03	13,399		12,784				Moderate
			lifetime prevalence of physician-diagnosed atopic eczema			10.1		13.8	P<0.001	Increase	
Haidinger et al. (2008) [57]	Austria (Upper)	6–7	ISAAC-based parental-report of:	1995–97/2001–03	35,238		12,541		% Change		Moderate
			lifetime prevalence of physician-diagnosed atopic eczema			9.9		13.6	3.7	Increase	
Weber et al. (2010) [63]	Austria (Upper)	6–9	ISAAC-based parental-report of:	1995–97/2001–03	12,115		11,468		No formal test		Moderate
			lifetime prevalence of physician-diagnosed atopic dermatitis			9.6		13.4		Increase	
Schernhammer et al. (2008) [61]	Austria (Upper)	12–14	ISAAC-based self-report of:	1995–97/2001–03	1,516		1,443				Moderate
			lifetime prevalence of physician-diagnosed atopic eczema			6.3		12.1	P<0.001	Increase	
Annesi-Maesano et al. (2009) [55]	France (Languedoc Roussillon)	13–14	ISAAC-based self-report of:	1995/2002	3,383		1,642		Absolute/relative % Change		Moderate
			lifetime prevalence of physician-diagnosed atopic dermatitis			25.8		30.4	4.56/0.17, P = 0.001	Increase	
**Southern Europe** *											
*Measures of symptoms of atopic eczema*											
Montefort et al. (2009) [64]	Maltese Islands	5–8	ISAAC-based parental-report of:	1994–95/2001–02	4,465		4,761				Moderate
			lifetime prevalence of recurrent rash			7.0		6.7	P = 0.61	Stable	
			12-month prevalence of recurrent rash			5.5		5.4	P = 0.85	Stable	
Galassi et al. (2006) [66]	Italy (North)	6–7	ISAAC-based parental-report of:	1994–95/2002	16,115		11,287		Area-adjusted absolute % Change (95% CI)		Good
			12-month prevalence of atopic eczema symptoms			8.3		14.5	6.2 (5.3–7.1)	Increase	
			12-month prevalence of atopic eczema symptoms in flexures			6.0		10.4	4.4 (3.6–5.2)	Increase	
Galassi et al. (2006) [66]	Italy (North)	13–14	ISAAC-based self-report of:	1994–95/2002	19,723		10,267		Area-adjusted absolute % Change (95% CI)		Good
			12-month prevalence of atopic eczema symptoms			10.1		11.2	1.2 (0.1–2.4)	Increase	
			12-month prevalence of atopic eczema symptoms in flexures			6.5		8.5	2.1 (1.2–3.0)	Increase	
Anthracopoulos et al. (2009) [65]	Greece (Patras)	8–10	Parental-report of:	1991/2003	2,417		2,725				Moderate
			lifetime prevalence of atopic eczema symptoms			4.5		9.5	P trend <0.001	Increase	
			24-month prevalence of atopic eczema symptoms			2.5		5.0	P trend <0.001	Increase	
*Measures of physician-diagnosed atopic eczema*											
Montefort et al. (2009) [64]	Maltese Islands	5–8	ISAAC-based parental-report of:	1994–95/2001–02	4,465		4,761				Moderate
			lifetime prevalence of physician-diagnosed atopic eczema			4.4		11.2	P<0.0001	Increase	
Galassi et al. (2006) [66]	Italy (North)	6–7	ISAAC-based parental-report of:	1994–95/2002	16,115		11,287		Area-adjusted absolute % Change (95% CI)		Good
			lifetime prevalence of atopic eczema			14.3		17	2.5 (1.6–3.5)	Increase	
Galassi et al. (2006) [66]	Italy (North)	13–14	ISAAC-based parental-report of:	1994–95/2002	19,723		9,362		Area-adjusted absolute % Change (95% CI)		Good
			lifetime prevalence of atopic eczema			11.0		12.8	1.5 (0.3–2.8)	Increase	
Rosado-Pinto et al. (2006) [67]	Portugal	6–7	ISAAC-based report of:	1993–94/2002	5,000		5,350				Moderate
			lifetime prevalence of atopic eczema			18.6		21.0	P = 0.002	Increase	
			12-month prevalence of atopic eczema			13.9		15.6	P = 0.013	Increase	
Rosado-Pinto et al. (2006) [67]	Portugal	13–14	ISAAC-based report of:	1993–94/2002	11,400		11,850				Moderate
			lifetime prevalence of atopic eczema			12.8		13.3	P = 0.22	Stable	
			12-month prevalence of atopic eczema			7.6		8.7	P = 0.002	Increase	
**Northern Europe** *											
*Measures of symptoms of atopic eczema*											
Annus et al. (2005) [69]	Estonia (Tallinn)	6–7	ISAAC-based parental-report of:	1993–94/2001–02	3,070		2,383		Sex-adjusted POR (95% CI)		Good
			lifetime prevalence of itchy rash			16.9		22.0	1.40 (1.22–1.61), P<0.001	Increase	
			12-month prevalence of itchy rash			12.6		17.1	1.44 (1.24–1.67), P<0.001	Increase	
			12-month prevalence of flexural rash			12.0		13.5	1.20 (1.02–1.41), P = 0.025	Increase	
Annus et al. (2005) [69]	Estonia (Tallinn)	13–14	ISAAC-based parental-report of:	1993–94/2001–02	3,476		3,576		Sex-adjusted POR (95% CI)		Good
			lifetime prevalence of itchy rash			15.2		19.3	1.34 (1.18–1.52), P<0.001	Increase	
			12-month prevalence of itchy rash			10.4		14.9	1.51 (1.31–1.74), P<0.001	Increase	
			12-month prevalence of flexural rash			7.7		9.4	1.26 (1.07–1.50), P = 0.006	Increase	
Shamssain et al. (2007) [80]	UK (North-east England)	6–7	ISAAC-based parental-report of:	1995–96/2001–02	3,000		1,843		OR (95% CI)		Good
			lifetime prevalence of rash in boys			17.8		21.0	1.6 (1.29–1.98)	Increase	
			lifetime prevalence of rash in girls			18.7		22.5	1.8(1.35–2.30)	Increase	
			lifetime prevalence of rash with typical distribution in boys			13.2		21.1	1.9 (1.41–3.57)	Increase	
			lifetime prevalence of rash with typical distribution in girls			14.7		23.8	1.8(1.35–2.25)	Increase	
			12-month prevalence of current rash in boys			14.7		23.3	1.4 (1.31–1.61)	Increase	
			12-month prevalence of current rash in girls			16.9		25.0	1.8(1.42–2.28)	Increase	
Shamssain et al. (2007) [80]	UK (North-east England)	13–14	ISAAC-based parental-report of:	1995–96/2001–02	3,000		2,195		OR (95% CI)		Good
			lifetime prevalence of rash in boys			13.9		15.3	1.1 (0.88–1.22)	Stable	
			lifetime prevalence of rash in girls			22.8		17.5	1.6 (1.29–1.98)	Increase	
			lifetime prevalence of rash with typical distribution in boys			8.8		19.6	2.4 (1.81–3.37)	Increase	
			lifetime prevalence of rash with typical distribution in girls			15.9		19.3	1.5 (1.12–1.98)	Increase	
			12-month prevalence of current rash in boys			11.3		16.8	1.6 (1.30–2.20)	Increase	
			12-month prevalence of current rash in girls			20.5		20.9	1.0 (0.89–1.32)	Stable	
Anderson et al. (2004) [68]	UK (British Isles)	12–14	ISAAC-based self-report of:	1995/2002	15,083		15,755		Absolute/Relative % Change		Moderate
			12-month prevalence of flexural rash			16.2		11.4	–4.8/−29.6	Decrease	
Bjerg et al. (2010) [70]	Sweden (Kiruna, Luleå, Piteå)	7–8	ISAAC-based parental-report of:	1996/2006	3,430		2,585				Good
			12-month prevalence of atopic eczema symptoms			27.2		25.8	P = 0.215	Stable	
Rönmark et al. (2009) [78]	Sweden (northern)	7–8	ISAAC-based parental-report of:	1996/2006	2,148		1,700				Moderate
			lifetime prevalence of atopic eczema symptoms			29.3		26.5	P = 0.048	Decrease	
Kudzytė et al. (2008) [72]	Lithuania (Kaunas)	6–7	ISAAC-based parental-report of:	1994–95/2001–02	1,879		2,772				Moderate
			12-months prevalence of itchy rash			2.6		3.9	P<0.05	Increase	
*Measures of physician-diagnosed atopic eczema*											
Kuehni et al. (2001) [63]	UK (Leicestershire)	1–5	Secondary analysis of:	1990/1998	1,264		2,127		Age- and sex-adjusted OR (95% CI)		Good
			lifetime prevalence of physicians’ recorded atopic eczema diagnosis			29.0		44.0	1.95 (1.68–2.27), P<0.001	Increase	
Shamssain et al. (2007) [80]	UK (North-east England)	6–7	ISAAC-based parental-report of:	1995–96/2001–02					OR (95% CI)		Good
			lifetime prevalence of atopic eczema in boys		1,445	27.8	918	37.0	1.9 (1.45–3.55), P = 0.001	Increase	
			lifetime prevalence of atopic eczema in girls		1,545	27.0	925	35.5	1.8 (1.45–2.45), P = 0.001	Increase	
Shamssain et al. (2007) [80]	UK (North-east England)	13–14	ISAAC-based parental-report of:	1995–96/2001–02					OR (95% CI)		Good
			lifetime prevalence of atopic eczema in boys		1,510	13.9	1,000	27.2	6.13 (3.52–10.79), P = 0.001	Increase	
			lifetime prevalence of atopic eczema in girls		1,490	22.8	1,195	30.7	1.63 (1.48–1.81), P = 0.001	Increase	
Ng Man Kwong et al. (2001) [76]	UK (Sheffield)	8–9	ISAAC-based parental-report of:	1991/1999	4,523		4,809		Absolute % Change (95% CI)		Moderate
			lifetime prevalence of atopic eczema			18.1		31.1	13.0 (11.27–14.72), P<0.001	Increase	
Anderson et al. (2004) [68]	UK (British Isles)	12–14	ISAAC-based self-report of:	1995/2002	15,083		15,755		Absolute/Relative % Change		Moderate
			lifetime prevalence of atopic eczema			21.1		24.3	3.3/15.4	Increase	
Simpson et al. (2009) [25]	UK	all	Secondary analysis of:	2001–2005	>9 million		>9 million		Relative % Change		Moderate
			age-and sex-standardised lifetime prevalence of physicians’ recorded atopic eczema diagnosis			7.8 (7.8–7.8)		11.5 (11.5–11.6)	48.2, P<0.001	Increase	
McNeill et al. (2009) [75]	Scotland (Aberdeen)	7–9	ISAAC-based parental-report of:	1999/2004	2,340	24.0 (22.3–25.7)	1,070	34.6 (32.3–36.9)	No formal test	Increase	Moderate
			lifetime prevalence of atopic eczema								
Osman et al. (2007) [77]	Scotland (Aberdeen)	9–11	ISAAC-based parental-report of:	1994/2004							Moderate
			lifetime prevalence of atopic eczema in boys		2,021	17.9	935	23.6	P trend<0.0001	Increase	
			lifetime prevalence of atopic eczema in girls		2,026	17.5	980	28.9	P trend<0.0001	Increase	
McNeill et al. (2009) [75]	Scotland (Aberdeen)	9–12	ISAAC-based parental-report of:	1999/2004	3,280		1,498		No formal test		Moderate
			lifetime prevalence of atopic eczema			21.1 (19.7–22.5)		34.2 (31.8–36.6)		Increase	
Devenny et al. (2004) [71]	Scotland (Aberdeen)	9–12	ISAAC-based parental-report of:	1994/1999	4,047		3,537		RR (95% CI)		Moderate
			lifetime prevalence of atopic eczema			18.0		21.0	1.2 (1.10–1.33)	Increase	
Kudzytė et al. (2008) [72]	Lithuania (Kaunas)	6–7	ISAAC-based parental-report of:	1994–95/2001–02	1,879		2,772				Moderate
			lifetime prevalence of physician-diagnosed atopic eczema			1.4		3.5	P<0.05	Increase	
Selnes et al. (2005) [79]	Norway (subarctic)	9–11	ISAAC-based self-report of:	1995/2000	1,432		3,853		RR (95% CI)		Moderate
			lifetime prevalence of atopic eczema			21.1		20.8	0.99 (0.88–1.11)	Stable	
Bjerg et al. (2010) [70]	Sweden (Kiruna, Luleå, Piteå)	7–8	ISAAC-based parental-report of:	1996/2006	3,430		2,585				Moderate
			lifetime prevalence of physician-diagnosed atopic eczema			13.4		15.2	P = 0.048	Increase	
Latvala et al. (2005) [74]	Finland	18–19	Report of:	1990–2000	–		–		No formal test		Moderate
			12-month prevalence of physician-diagnosed atopic eczema			1.2#		1.2#		Stable	
**Eastern Europe** *											
*Measures of physician-diagnosed atopic eczema*											
Harangi et al. (2007) [82]	Hungary (Baranya County)	7–9	Hanifin-Rajka criteria-based parental-report of:	2002/2005	587		574		No formal test		Moderate
			physician-diagnosed atopic dermatitis			17.0		17.1		Stable	
Harangi et al. (2007) [82]	Hungary (Baranya County)	7–14	Hanifin-Rajka criteria-based parental-report of:	2002/2005	1,454		1,454		No formal test		Moderate
			physician-diagnosed atopic dermatitis			15.1		16.1		Stable	
Brożek et al. (2004) [81]	Poland (Chorzów)	7–10	Parental-report of:	1993/2002	1,130		1,451				Moderate
			lifetime prevalence of physician-diagnosed atopic eczema			2.3		8.1	P<0.001	Increase	

Abbreviations – CI: confidence intervals, SE: standard error, POR: prevalence odds ratio, OR: odds ratio.

* Based on UN classification [16].

** 95% CI and SE are only reported if included in original report.

# Point estimate extracted from graph or chart.

In southern Europe, the lifetime prevalence of atopic eczema symptoms remained stable in the Maltese Islands in 5–8 year olds [7.0% (1994–95) and 6.7% (2001–02)] [64] and increased in Greece in older children (aged 8–10) [from 4.5% (1991) to 9.5% (2003)] [65]. Here, the trend was measured over a longer time period and started at a lower baseline level. In Italy and Portugal, no lifetime prevalence trends for atopic eczema symptoms were reported. The lifetime prevalence of physician-diagnosed atopic eczema showed an increasing trend in 6–7 year olds in Italy [from 14.3% (1994–95) to 17.0% (2002)] [66] and in the same age group in Portugal [from 18.6% (1993–94) to 21.0% (2002)] [67]. Compared to Italy and Portugal, the increase of physician-diagnosed atopic eczema in the Maltese Islands in a similar age group of 5–8 year olds was considerably larger and more than doubled over a 7-year period, as it originated from a low baseline [from 4.4% (1994–95) to 11.2% (2001–02)] [64].

For northern Europe, 15 papers reported on trends in atopic eczema [23], [25], [68]–[80]. The prevalence of rash and rash with a typical distribution was overall increasing in boys and girls and in 6–7 and 13–14 year olds in the UK [e.g. in 6–7 year old boys – from 17.8% (1995–96) to 21.0% (2001–02)], although not all trends reached significance [80]. Several other studies, which measured the lifetime prevalence of physician-diagnosed atopic eczema [68], [71], [75]–[77] or the lifetime prevalence of physicians’ recorded atopic eczema diagnosis [25], [73] in the UK, also showed increasing trends confirming patterns of atopic eczema prevalence in the UK in children and across all age-groups over time. An increasing trend for atopic eczema symptoms was also found in Estonia in 6–7 year olds [from 16.9% (1993–94) to 22.0% (2001–02)] [69]. However, in Sweden in slightly older children (aged 7–8), the lifetime prevalence of atopic eczema symptoms was decreasing [from 29.3% (1996) to 26.5% (2006)] [78]. Despite this decrease, prevalence estimates remained higher as compared to Estonia. There was no data on the lifetime prevalence of atopic eczema symptoms available in Lithuania and Norway. In Lithuania in 6–7 year olds, the prevalence of physician-diagnosed atopic eczema was increasing from an extremely low baseline [from 1.4% (1994–95) to 3.5% (2002–03)] [72]. In Norway, this prevalence was stable in 9–11 year olds [21.1% (1995) and 20.8% (2000)] [79].

Two studies yielded relevant data in relation to eastern Europe. In Poland, the lifetime prevalence of parental-reported physician-diagnosed atopic eczema increase over a decade in children aged 7–10 [from 2.3% (1993) to 8.1% (2002)] [81]. Later, this same prevalence, measured with a different questionnaire in both 7–9 and 7–14 year olds, remained stable in Hungary over a relatively short time period [e.g. for 7–9 year olds –15.1% (2002) and 17.1% (2005)] [82]. In Europe, there were many other countries with single measurements of any atopic eczema outcome, but serial data were not yet available.

### Oceania

For Oceania, we found three papers from Australia with prevalence data on atopic eczema trends (see Table 4) [83]–[85]. As shown in Table 10, the lifetime prevalence of atopic eczema symptoms was measured in Melbourne in 6–7 year olds, where it markedly increased from 22.6% in 1993 to 32.3% in 2002 [84]. Two other studies measured trends in lifetime prevalence of atopic eczema diagnosis. In one study this was increasing in 4–6 year olds, even though the baseline prevalence was high [from 31.0% (2000) to 37.0% (2005)] [83] and in another study, using a non-validated questionnaire, it was stable in 8–11 year olds [85].

**Table 10:** Good and moderate quality studies reporting the prevalence of parental- or self-report of atopic eczema between 1990 and 2010 in Oceania.

Study	Geographic area	Age range	Outcome	Time period	Baseline estimate		Final estimate		Summary measures	Conclusion	Quality
		(y)			N	% (95%CI)/(SE)**	N	% (95%CI)/(SE)**			
*Measures of symptoms of atopic eczema*											
Robertson et al. (2004)[84]	Australia (Melbourne)	6–7	ISAAC-based parental-report of:	1993/2002	2,843		2,968		No formal test		Moderate
			lifetime prevalence of atopic eczema symptoms			22.6 (20.8–24.6)		32.3 (30.4–34.2)		Increase	
			12-month prevalence of atopic eczema symptoms			11.1 (10.0–12.3)		17.2 (15.7–18.8)		Increase	
*Measures of physician-diagnosed atopic eczema*											
Ponsonby et al. (2008) [83]	Australia (Australian Capital Territory)	4–6	Annual school entry-based and ISAAC-based parental-report of:	2000–05	3,873		3,849		Adjusted OR (95% CI) per year		Good
			lifetime prevalence of atopic eczema			31.0#		37.0#	1.05 (1.03–1.07) P<0.001	Increase	
Toelle et al. (2004) [85]	Australia (Belmont)	8–11	ISAAC-based parental-report of:	1992/2002	908		800		% Change (95%CI)		Moderate
			lifetime prevalence of atopic eczema			24.4		24.8	0.4 (−3.7–4.5), P = not significant	Stable	

Abbreviations – CI: confidence intervals, SE: standard error, OR: odds ratio.

* Based on UN classification [16].

** 95% CI and SE are only reported if included in original report.

# Point estimate extracted from graph or chart.

## Discussion

The considerable body of international literature identified by this systematic review was heterogeneous in many respects rendering it difficult to directly compare different regions. That said, there was no obvious consistent overall global trend in the incidence or prevalence of atopic eczema symptoms and diagnosis. Nevertheless, in Africa and eastern Asia there was an increasing trend for both the lifetime prevalence of parental- and self-reported atopic eczema symptoms and physician-diagnosed atopic eczema. In western Europe and parts of northern Europe (i.e. the UK), these trends were also mainly increasing. There were extremely diverse trends among different age groups and countries in south-eastern Asia, western Asia and southern Europe. In addition, data for the Americas, eastern Europe and Oceania were limited. The heterogeneous findings in some regions and the limited data available for other regions have precluded conclusions regarding a global atopic eczema trend and atopic eczema trends in major parts of the world.

We found that many outcome measures are used across studies to determine changes in atopic eczema prevalence. Although we found that trends of all outcomes generally pointed in the same direction, we considered the lifetime prevalence of parental- or self-report of atopic eczema symptoms the optimal outcome for the purpose of comparing disease trends between regions within our highly heterogeneous dataset. As atopic eczema occurs in episodes and may be season-related it is particularly difficult to compare studies measuring current or 12-month symptomatology or if patient- and/or study- characteristics, such as age group, do not match. Furthermore, there are marked differences per region in current medical practice, including prevention strategies, national guidelines and physician’s awareness of the problem, that make prevalence estimates and trends of physician-diagnosed atopic eczema difficult to compare across the globe. Even though the diagnostic process of a physician is overall likely to be standardised, there is no objective gold standard. This is highlighted in the ENRIECO project which shows that different countries use different terms to describe atopic eczema [86]. In addition, not every language has disease labels, nor are they understood in the same way. This means that a diagnostic label may be influenced by region-specific guidelines for the diagnosis of atopic eczema and this may therefore render it difficult to compare estimates of physician-diagnosed atopic eczema prevalence between regions. We thus judged that the lifetime prevalence of atopic eczema symptoms was most likely to prove useful in relation to yielding comparative data on trends in atopic eczema.

### 

#### Strengths and limitations

To our knowledge, no systematic review on international disease trends in the incidence and prevalence of atopic eczema has been published. We searched a large amount of potential relevant literature using seven electronic databases and included 69 papers which reported on trends in atopic eczema. These should represent a good coverage of published literature. Furthermore, we searched systematically, according to a protocol and used stipulated inclusion criteria. To ensure that included studies are above a specific quality threshold, the studies were independently quality-filtered by two reviewers. Where a consensus could not be obtained a third reviewer provided arbitration guidance. In contrast with earlier work into this field, we included all reports on atopic eczema trends, whereas previously papers have limited themselves to single estimates of atopic eczema [4], [5], or to original data from the ISAAC study [2], [87].

There are gaps in the literature. We could include particularly few reports from the Americas, eastern Europe and Oceania. In general, studies are available on the prevalence of atopic eczema in these regions. However, information from these studies will not be relevant until they are repeated over time. This perhaps somewhat surprising gap for North America is likely to be, at least in part, due to the fact that the ISAAC programme had difficulty identifying a regional coordinator for this region [88]. We were unable to obtain the full-text translation of one Korean paper. Nevertheless, we are reasonably confident that this report or any other additional reports would be unlikely to undermine our overall findings – that there is no clear trend in the worldwide incidence and prevalence of atopic eczema. For nearly all regions information on atopic eczema is questionnaire-based. Questionnaires are non-specific and the measured symptoms suggestive of atopic eczema may overlap with symptoms of other conditions, such as contact dermatitis. The ISAAC questionnaire stipulates the typical distribution and the onset of the itchy rash (see Table 2), which helps to enhance its specificity. At the population level and particularly for the purpose of between-population comparison, ISAAC questions are therefore likely to provide adequate symptom-derived prevalence estimates [89]. That said there is inevitably some loss of ability to differentiate between atopic eczema and other differential diagnoses such as allergic contact dermatitis. This problem may have been more pronounced had we also identified studies using the ECRHS; in the event however, no such relevant studies were found to be eligible.

#### Future work

Further research in this area should firstly address methodological issues to help inform the optimum design, execution and reporting of future epidemiological studies of trends in atopic eczema. In our dataset various outcome measures were reported and various assessment tools were used, data were analysed differently across studies and results were reported in different formats (e.g. with and without confidence intervals (CI)), age groups did not match and studies were inconsistently stratified for sex. All of these factors enhance the incomparability of studies. In view of the above, we suggest full and elaborate reporting of the results (including CI) of all of the outcomes obtained. We recommend that the above gaps be addressed using the complete ISAAC tool (and, where possible, also include detailed clinical assessment to allow atopic eczema to be differentiated from allergic contact dermatitis) and be reported according to a standardised format, so that comparisons to other reports on trends are possible. However, even if studies are comparable the prevalence of atopic eczema may still be difficult to compare across countries, without a universal definition. Thus, we need a range of relevant measures of incidence and prevalence as well as a careful description of the diagnostic criteria used together with appropriate interpretation of these data in order to ensure that this important field of epidemiological enquiry progresses in a scientifically robust manner.

#### Interpretation

Although there is no consistent overall global trend in atopic eczema incidence and prevalence, there are some specific trends which are worth remarking upon further, as they may be of interest for research into the aetiology of atopic eczema. Firstly, there was a stable incidence of atopic eczema in 5–6 year olds in West Germany [12.5% (1991) and 12.8% (1997)] and a sharply increasing incidence in East Germany [from 9.6% (1991) to 23.4% (1997)] [24]. This coincides with the adoption of a “Western” lifestyle in East Germany as a result of political change. A consequence of changed lifestyle and increased socio-economic wealth may be an increased frequency of bathing and a greater availability of soaps and bubble baths, which may remove the skin’s natural barrier oils and make atopic eczema more prevalent [90]. This is a biologically credible mechanism to explain an increase in incidence – in particular of mild disease. Nevertheless, Schafer et al. (2000) found that, after adjustment for potential confounders, including socio-economic status, the difference in incidence between East and West Germany remained [24]. Other factors, such as nutritional factors, allergens and irritants or infections may therefore be important in the aetiology of atopic eczema. Political changes resulting in, for example, improved access to physicians in East Germany after reunification or due to changes in disease labelling could also have impacted on measures of the incidence of atopic eczema, as has been noted in relation to asthma diagnosis and prescribing [91]. If this were the case, this would reflect an increase in reporting behaviour rather than any true change in the epidemiology of eczema.

Other trends of interest regarding aetiological hypotheses include diverging trends between neighbouring regions. For example, there are marked increases in lifetime prevalence of atopic eczema symptoms in most countries in Africa [e.g. in South Africa – from 15.5% (1995) to 26.2% (2002)] [29], whereas there is a large decrease in Nigeria [from 26.1% (1995) to 18.0% (2001–02)] [27]. This anomalous decrease is most likely a consequence of the extremely high baseline prevalence, as prevalence estimates in 2001–02 are largely comparable for all African countries. Rather than a true prevalence, this high baseline estimate may be a reflection of the presence of another skin condition, such as another rash, perhaps caused by parasites, which are common in these regions. In addition, in our dataset there were also marked baseline differences between neighbouring countries. This is indicated by the low baseline prevalence of atopic eczema symptoms in 12–15 year olds in Taiwan [2.4% (1995–96)] [32] and the much higher baseline prevalence in Korea [7.2% (1995)] [36]. In these countries too, cultural, social and diagnostic differences may potentially explain this pattern. In contrast, large changes in prevalence estimates within one country in a short space of time are of interest as such changes are likely to represent a true change. For example the doubling in lifetime prevalence of both atopic eczema diagnosis [from 13.9% (1995–96) to 27.2% (2001–02)] and atopic eczema symptoms [from 8.8% (1995–96) to 19.6% (2001–02)] in boys aged 13–14 in England [80] is likely to represent a true change and we must consider environmental explanations for this.

In conclusion, we have found no overall trend for the incidence or prevalence of atopic eczema worldwide. However, in Africa, eastern Asia, western Europe and parts of northern Europe (i.e. the UK) trends in atopic eczema prevalence were mainly increasing. There are gaps in the literature, particularly in the Americas and Oceania and for measures of atopic eczema incidence. Future research should investigate trends in what is now one of the most prevalent disorders in Europe and other regions in a scientifically robust manner. In order to do so, the careful use of key definitions, improved study design and more comprehensive reporting are essential.

## Supporting Information

Appendix S1 **PRISMA checklist.** PRISMA checklist with 27 reporting items used for the systematic review.
(DOC)

Appendix S2 **Search terms.** Search terms and limitations used for the systematic review.
(DOC)
