# Supplementary material for: Investigating International Time Trends in the Incidence and Prevalence of Atopic Eczema 1990–2010: A Systematic Review of Epidemiological Studies
Source: PLoS One. 2012 Jul 11;7(7):e39803. doi: 10.1371/journal.pone.0039803 (PMC3394782; doi:10.1371/journal.pone.0039803)
Supplement: Appendix S2 — Search terms. Search terms and limitations used for the systematic review. (DOC) [file pone.0039803.s002.doc]

**Appendix S2: Search terms**

Medline (OVID), EMBASE (OVID)

1. Eczema/ ep [Epidemiology]
2. eczema
3. infantile eczema
4. eczema, infantile
5. 3 OR 4
6. flexural eczema
7. eczema, flexural
8. 6 OR 7
9. atopic eczema
10. eczema, atopic
11. 9 OR 10
12. 1 OR 2 OR 5 OR 8 OR 11
13. Dermatitis, Atopic/ep [Epidemiology]
14. dermatitis, atopic
15. atopic dermatitis
16. 13 OR 14 OR 15
17. 12 OR 16
18. exp Cohort studies/
19. Cohort.mp
20. 18 OR 19
21. Cross-sectional studies/
22. Cross-sectional
23. 21 OR 22
24. Questionnaires/
25. Questionnaires
26. Questionnaire
27. 24 OR 25 OR 26
28. Health Surveys/
29. surveys
30. survey
31. 28 OR 29 OR 30
32. ISAAC
33. ECRHS
34. 32 OR 33
35. 20 OR 23 OR 27 OR 31 OR 34
36. Incidence/
37. Incidence
38. 36 OR 37
39. Prevalence/
40. Prevalence
41. 39 OR 40
42. trend
43. trends
44. 42 OR 43
45. 38 OR 41 OR 44
46. 17 AND 35 AND 45

*Limited to publication date 1990-current and humans only*

CINAHL, Global Health, Global Health library and Google Scholar

((Eczema or infantile eczema or eczema, infantile or flexural eczema or eczema, flexural or atopic eczema or eczema, atopic) or (Dermatitis, Atopic or atopic dermatitis)) AND ((Cohort studies or Cohort or Cross-sectional studies or Cross-sectional or Questionnaires or Questionnaire or Health Surveys or surveys or survey) or (ISAAC or ECRHS)) AND (incidence or prevalence or trend or trends)

*Llimited to publication date 1990-current*
